# Maresin 1 Inhibits TRPV1 in Temporomandibular Joint-Related Trigeminal Nociceptive Neurons and TMJ Inflammation-Induced Synaptic Plasticity in the Trigeminal Nucleus

**DOI:** 10.1155/2015/275126

**Published:** 2015-11-05

**Authors:** Chul-Kyu Park

**Affiliations:** Department of Physiology, College of Medicine, Gachon University, Incheon 406-799, Republic of Korea

## Abstract

In the trigeminal system, disruption of acute resolution processing may lead to uncontrolled inflammation and chronic pain associated with the temporomandibular joint (TMJ). Currently, there are no effective treatments for TMJ pain. Recently, it has been recognized that maresin 1, a newly identified macrophage-derived mediator of inflammation resolution, is a potent analgesic for somatic inflammatory pain without noticeable side effects in mice and a potent endogenous inhibitor of transient receptor potential vanilloid 1 (TRPV1) in the somatic system. However, the molecular mechanisms underlying the analgesic actions of maresin 1 on TMJ pain are unclear in the trigeminal system. Here, by performing TMJ injection of a retrograde labeling tracer DiI (a fluorescent dye), I showed that maresin 1 potently inhibits capsaicin-induced TRPV1 currents and neuronal activity via G*α*i-coupled G-protein coupled receptors in DiI-labeled trigeminal nociceptive neurons. Further, maresin 1 blocked TRPV1 agonist-evoked increases in spontaneous excitatory postsynaptic current frequency and abolished TMJ inflammation-induced synaptic plasticity in the trigeminal nucleus. These results demonstrate the potent actions of maresin 1 in regulating TRPV1 in the trigeminal system. Thus, maresin 1 may serve as a novel endogenous inhibitor for treating TMJ-inflammatory pain in the orofacial region.

## 1. Introduction

Temporomandibular joint (TMJ) disorders refer to a heterogeneous group of clinical conditions including pain and limited movement of the TMJ. In addition to the increased pain sensitivity and referred pain beyond the affected TMJ, both autonomic and endocrine functions can be altered and TMJ inflammation can be persistent [1–3]. While the exact mechanisms of TMJ pain remain unclear, a growing body of evidence indicates that persistent TMJ pain conditions may be mediated by both peripheral and central mechanisms in the trigeminal nervous system.

Inflammation of the TMJ region is a likely triggering factor leading to the pathogenesis of persistent TMJ pain [4, 5], which may result in increased neuronal excitability in both the peripheral nervous system (e.g., trigeminal ganglion) and the central nervous system (e.g., trigeminal nuclei) [6, 7]. Transient receptor potential vanilloid 1 (TRPV1) and ankyrin 1 (TRPA1) are two critical types of TRP channels that are strongly implicated in the genesis of somatic inflammatory pain [8, 9]. Activation of TRPV1 and TRPA1 can enhance somatic inflammatory pain not only via peripheral sensitization [8, 10, 11], but also via central sensitization [12]. TRPV1 and TRPA1 have also been shown to be involved in persistent pain in the trigeminal system [13–16]. Great pharmaceutical effort has been devoted to developing small-molecule inhibitors of TRP channels (e.g., TRPV1 and TRPA1) but with limited success. However, little is known about the endogenous inhibitors of TRP channels. There are no effective treatments for chronic pain associated with TMJ.

It has been recently identified that several families of novel proresolution lipid mediators (PRLMs), such as resolvins and protectins, derived from omega-3 unsaturated fatty acids and further demonstrated these PRLMs possess potent anti-inflammatory and proresolution actions in animal models [17–19]. Maresins, derived from docosahexaenoic acid (DHA), are newly described macrophage-derived mediators of inflammation resolution [20]. The term maresin is coined from macrophage mediator in resolving inflammation. Maresins possess potent anti-inflammatory and proresolving properties [21]. DHA, added to activate macrophages, can be converted into maresins. Maresin 1 (7R, 14S-dihydroxy-docosa-4Z, 8E, 10E, 12Z, 16Z, 19Z-hexaenoic acid) is the first identified maresin family member [20]. Maresin 1 shows potent analgesic actions by inhibiting formalin-induced inflammatory pain and chemotherapy-induced neuropathic pain in the somatic system and suppressing TRPV1 activity in dorsal root ganglion (DRG) neurons [21]. However, the role of maresin 1 in regulating different TRP channels, synaptic transmission, and TMJ pain in the trigeminal system has yet to be examined. In the present study, I investigated whether and how maresin 1 can effectively modulate TRP channels in TMJ-related trigeminal primary afferent neurons and TMJ inflammation-induced synaptic plasticity in the trigeminal nucleus.

## 2. Materials and Methods

### 2.1. Animals

All surgical and experimental procedures were reviewed and approved by the Institutional Animal Care and Use Committee in College of Medicine, Gachon University. Adult C57BL/6 male mice were purchased from Orientbio (Sungnam, Korea). Animals were habituated for at least 1 week prior to experiments in a conventional facility with a 12:12 h light cycle (lights on 8.00 am) and had ad libitum access to water and food.

### 2.2. Preparation of Trigeminal Ganglion (TG) Neurons Innervating the Temporomandibular Joint (TMJ)

TMJ-related trigeminal ganglion neurons were identified by TMJ injection of a retrograde labeling tracer, DiI (D-282, Molecular Probes, Eugene, OR, USA), a fluorescent dye [22, 23]. Briefly, mice were anesthetized with 2% isoflurane. The skin overlying the TMJ was shaved, and the injection target was identified by palpating the zygomatic arch and mandible. The retrograde tracer DiI (3 *μ*L; 17 mg/mL) was slowly injected into the TMJ capsule. The needle (30-gauge) was left in place for ~2 minutes before being withdrawn in order to minimize leakage of dye from the injection site. After 3 days, trigeminal ganglia prepared in 4°C Hank's Balanced Salt Solution (HBSS; Welgene, Daegu, Korea) were incubated in 2 mL HBSS containing 0.25% trypsin (Invitrogen, Carlsbad, CA, USA) at 37°C for 60 min. The cells were washed, triturated with a flame-polished Pasteur pipette, and placed on 0.5 mg/mL poly-L-ornithine (Sigma, St. Louis, MO, USA) coated glass coverslips. The cells were maintained at 37°C in a 5% CO_2_ incubator.

### 2.3. Single-Cell Reverse-Transcription Polymerase Chain Reaction (RT-PCR)

Single-cell RT-PCR was performed as previously described [24]. Briefly, following whole-cell patch clamp recordings, DiI-labeled TG neurons were harvested into patch pipettes with tip diameters of about 15–25 *μ*m, gently put into reaction tubes containing reverse-transcription reagents, and incubated for 1 h at 50°C (Superscript III, Invitrogen). The cDNA products were used in separate PCRs. The sequences of the primers used are shown in Table 1. The first round of PCR was performed in 50 *μ*L of PCR buffer containing 0.2 mM dNTPs, 0.2 *μ*M “outer” primers, 5 *μ*L RT product, and 0.2 *μ*L platinum Taq DNA polymerase (Invitrogen). The protocol included an initial 5 min denaturizing step at 95°C followed by 40 cycles of 40 s denaturation at 95°C, 40 s annealing at 55°C, and 40 s elongation at 72°C. The reaction was completed with 7 min of final elongation. For the second round of amplification, the reaction buffer (20 *μ*L) contained 0.2 mM dNTPs, 0.2 *μ*M “inner” primers, 5 *μ*L of the first round PCR products, and 0.1 *μ*L platinum Taq DNA polymerase. The amplification procedure for the inner primers was the same as that for the first round. A negative control was obtained from pipettes that did not have cell contents but were submerged in the bath solution. The PCR products were displayed on ethidium bromide-stained agarose gels (2%).

### 2.4. Whole-Cell Patch Clamp Recordings in DiI-Labeled TG Neurons

DiI-labeled TG neurons were visualized using a fixed-stage fluorescence microscope (BX50WI, Olympus, Japan) with Nomarski optics and the sizes of soma were estimated. As previously described [22], DiI-labeled TG neurons were classified into three groups, small-sized (0–22 *μ*m), medium-sized (23–37 *μ*m), and large-sized (38–60 *μ*m) neurons. Whole-cell voltage- and current-clamp recordings were performed at room temperature to measure currents and action potentials, respectively, with an Axopatch-200B amplifier (Axon Instruments, Union City, CA, USA). The patch pipettes were pulled from borosilicate capillaries (Chase Scientific Glass Inc., Rockwood, TN, USA). When filled with the pipette solution, the resistance of the pipettes was 4-5 MΩ. The recording chamber (300 *μ*L) was continuously superfused (2-3 mL/min). Series resistance was compensated for (>80%), and leak subtraction was performed. Data were low-pass-filtered at 2 kHz and sampled at 10 kHz. The pClamp8 (Axon Instruments) software was used during experiments and analysis. The pipette solution for voltage-clamp experiments was composed of (in mM) 126 K-gluconate, 10 NaCl, 1 MgCl_2_, 10 EGTA, 2 NaATP, and 0.1 MgGTP, adjusted to pH 7.4 with KOH and osmolarity 295–300 mOsm. Ca^2+^-free extracellular solution contained 0 mM CaCl_2_ and 2 mM EGTA for chelation of ambient Ca^2+^. The extracellular solution for voltage-clamp experiments contained (in mM) 140 NaCl, 5 KCl, 2 CaCl_2_, 1 MgCl_2_, 10 HEPES, and 10 glucose, adjusted to pH 7.4 with NaOH and osmolarity 300–310 mOsm. Voltage-clamp experiments were performed at a holding potential of −60 mV.

### 2.5. Trigeminal Nucleus Slice Preparation and Patch Clamp Recordings

A portion of the subnucleus caudalis (Sp5C) was removed from mice (4–6 weeks old) under urethane anesthesia (1.5–2.0 g/kg, i.p.) and kept in preoxygenated ice-cold Kreb's solution. Transverse slices (200–400 *μ*m) were cut on a vibrating microslicer. The slices were perfused with Kreb's solution (8–10 mL/min) and saturated with 95% O_2_ and 5% CO_2_ at 37°C. Whole-cell patch clamp recordings were made from the superficial layers of the Sp5C (lamina I-II) neurons in voltage-clamp mode at 37°C. After establishing the whole-cell configuration, neurons were held at the potential of −70 mV to record sEPSCs. The internal solution contained (in mM) 135 K-gluconate, 5 KCl, 0.5 CaCl_2_, 2 MgCl_2_, 5 EGTA, 5 HEPES, and 5 ATP-Mg, adjusted to pH 7.4 (with KOH) and osmolarity 295–300 mOsm. The perfusion solution contained (in mM) 117 NaCl, 3.6 KCl, 2.5 CaCl_2_, 1.2 MgCl_2_, 1.2 NaH_2_PO_4_, 25 NaHCO_3_, and 11 glucose. The resistance of a typical patch pipette is 5–10 MΩ. Membrane currents were amplified with an Axopatch 200B amplifier (Axon Instruments) in voltage-clamp mode. Signals were filtered at 2 kHz and digitized at 5 kHz. Data were stored on a personal computer using pCLAMP 10 software and analyzed with Mini Analysis (Synaptosoft Inc., Fort Lee, NJ, USA).

### 2.6. Drugs and Administration

Capsaicin and allyl isothiocyanate (AITC), complete Freund's adjuvant (CFA), were obtained from Sigma. Maresin 1 was purchased from Cayman Chemical. Pertussis toxin (PTX), an irreversible inhibitor of G*α*i-coupled G-protein coupled receptors (GPCRs), was purchased from Tocris Bioscience (Bristol, UK). To determine the involvement of GPCRs in maresin 1′ actions, DRG neurons were cultured with a PTX (0.5 *μ*g/mL) for 18 h. Unilateral inflammation was induced by injecting CFA (20 *μ*L, 1 mg/mL) into the TMJ using a 30-gauge needle [23]. The injection site was identified as described for the retrograde tracer DiI injection.

### 2.7. Statistical Analysis

All data are expressed as mean ± SEM. ANOVA or Student's *t*-test was used to determine the differences using the software Origin 6.0 (Microcal Software, Inc., Northampton, MA, USA). Differences were considered to be significant when *p* value was less than 0.05.

## 3. Results

### 3.1. Distribution of DiI-Labeled Trigeminal Primary Afferent Neurons

**Figure 1:**
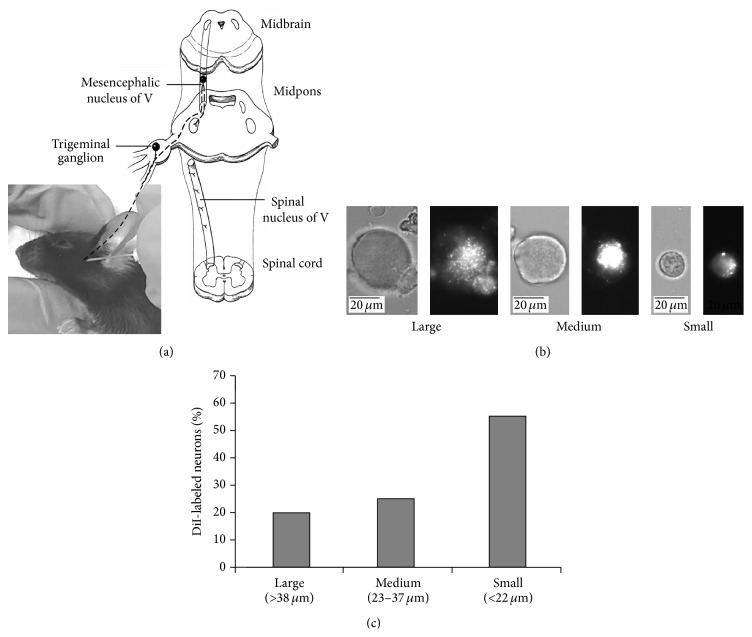
Verification of DiI-labeled trigeminal primary afferent neurons. (a) Retrograde labeling of trigeminal primary afferent neurons by TMJ injection with the fluorescent dye DiI. (b) Identification of DiI-labeled dissociated TG neurons. As illustrated, the representative large, medium, and small neurons are visualized under phase-contrast (left), and fluorescent (right) images, respectively. (c) The proportion of DiI-labeled dissociated TG neurons by diameter.

The trigeminal ganglion (TG) is the location of primary afferent neurons for sensing and relaying nociceptive sensations associated with painful conditions such as dental pain, trigeminal neuralgia, and TMJ pain [25–27]. I labeled the TG neurons by TMJ injection of a retrograde labeling tracer, DiI, a fluorescent dye [22], as shown in Figure 1(a). Three days later, DiI labeling was detected in the dissociated TG neurons (Figure 1(b)), indicating that trigeminal primary afferent neurons innervating the TMJ can be investigated* in vitro*. Interestingly, the proportion of DiI-labeled neurons is relatively higher in small-sized neurons (*n* = 99/180, 55%), compared with medium-sized neurons (*n* = 45/180, 25%) and large-sized neurons (*n* = 36/180, 20%) (Figure 1(c)). These data demonstrate that DiI-labeled small-sized TG neurons are responsible for pain sensation as nociceptors associated with TMJ pain [22, 24].

### 3.2. Maresin 1 Inhibits TRPV1 Activity in DiI-Labeled TG Neurons via G*α*i-Coupled G-Protein Coupled Receptors (GPCRs)

**Figure 2:**
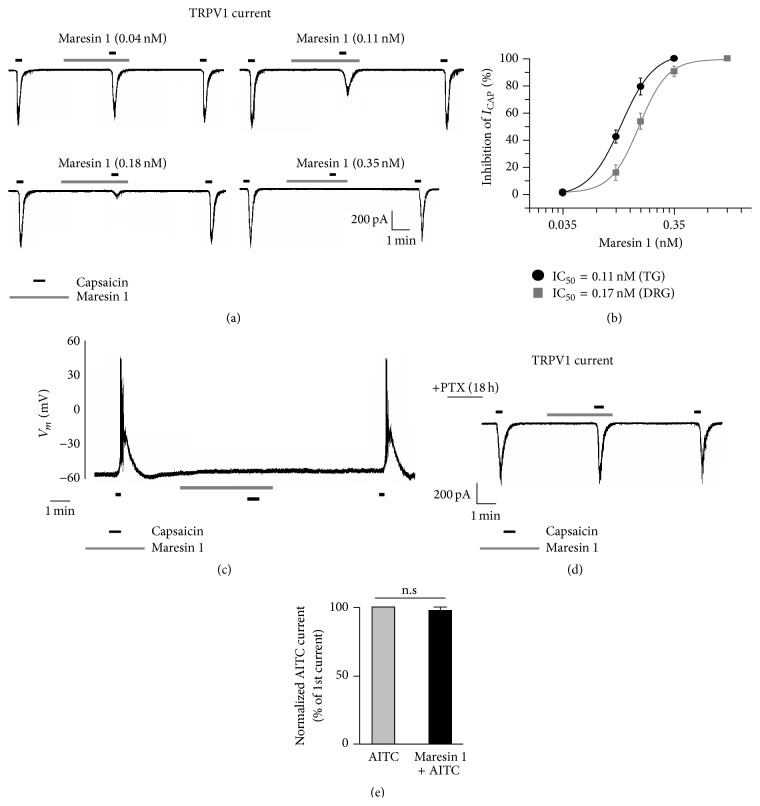
Maresin 1 potently inhibits TRPV1 but not TRPA1 current in DiI-labeled small-sized TG neurons. (a) Voltage-clamp recordings showing dose-dependent inhibition of capsaicin-induced (100 nM) TRPV1 currents by maresin 1 in DiI-labeled small-sized neurons. *n* = 8 for each dose. (b) Dose response curve of maresin 1-induced inhibition of TRPV1 currents in DiI-labeled small-sized neurons (circle, black line) and in small-sized DRG neurons (square, gray line). Inset: IC_50_ of TRPV1 current inhibition in TG and DRG neurons, respectively. (c) Current-clamp recording showing blockade of capsaicin-induced action potentials by maresin 1 (0.35 nM), *n* = 15. (d) PTX pretreatment (0.5 *μ*g/mL, 18 h) abolishes the inhibitory effects on maresin 1 on TRPV1 currents, *n* = 15. (e) Maresin 1 (0.35 nM) does not inhibit AITC-induced (300 *μ*M) TRPA1 currents (*p* > 0.05 and *n* = 15, *t*-test).

By performing patch clamp recordings in DiI-labeled small-sized TG neurons, I investigated whether maresin 1 regulates TRPV1 and TRPA1 activity. Perfusion of DiI-labeled small-sized TG neurons with capsaicin (100 nM) elicited a marked TRPV1 current, and this current was dose-dependently inhibited by maresin 1 (Figure 2(a)). Notably, maresin 1 inhibited TRPV1 currents with a very low IC_50_ (0.11 nM) in DiI-labeled small-sized TG neurons. For comparison, maresin 1 also inhibited TRPV1 currents with an IC_50_ (0.17 nM) in small-sized DRG neurons, indicating that the IC_50_ of maresin 1 for inhibiting TRPV1 currents in TG neurons is lower than that in DRG neurons (Figure 2(b)). In addition, current-clamp recording revealed that maresin 1 also completely blocked capsaicin-induced action potentials (Figure 2(c)). Notably, pretreatment of DiI-labeled small-sized neurons with pertussis toxin (PTX, 0.5 *μ*g/mL) for 18 h completely blocked the inhibitory effects of maresin 1 on capsaicin-induced TRPV1 currents (Figure 2(d)), suggesting a G*α*i-coupled GPCR pathway. Interestingly, the highest concentration of maresin 1 (0.35 nM) failed to inhibit the AITC-induced TRPA1 current in DiI-labeled small-sized neurons (Figure 2(e)).

### 3.3. TRPV1 and Na_v_1.8 Are Expressed in DiI-Labeled Small-Sized TG Neurons

**Figure 3:**
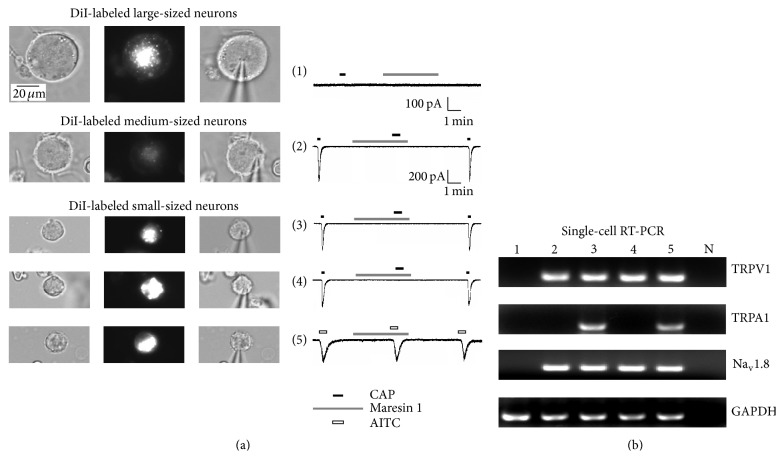
Voltage-clamp recordings and single-cell RT-PCR in five DiI-labeled TG neurons. (a) Representative DiI-labeled large-, medium-, and small-sized TG neurons are visualized under phase-contrast (left) and fluorescent (middle) images, respectively. The right image shows the whole-cell patch clamp recording mode in DiI-labeled neurons. Capsaicin (CAP, 100 nM) did not induce inward currents in DiI-labeled large-sized neuron (#1). Three neurons (#2, #3, and #4) responded to capsaicin with inward currents, which were completely blocked by maresin 1 (0.35 nM). Note that maresin 1 (0.35 nM) did not inhibit AITC-induced (300 *μ*M) TRPA1 currents in DiI-labeled small-sized neuron (#5). (b) Single-cell RT-PCR analysis following voltage-clamp recording in five DiI-labeled TG neurons. GAPDH is the positive control. *N* is the negative control.

The nociceptive markers, TRPV1 and Na_v_1.8, are known to contribute to pain transduction in nociceptive neurons [22, 24]. Therefore, I analyzed expression of TRPV1 and Na_v_1.8 mRNAs using single-cell RT-PCR following electrophysiological and pharmacological characterization of DiI-labeled TG neurons by whole-cell recordings. Maresin 1 (0.35 nM) completely blocked capsaicin-induced inward currents in DiI-labeled medium- and small-sized neurons (Figure 3(a); cells #2, #3, and #4), and consistently, these three neurons expressed TRPV1 and Na_v_1.8 mRNAs (Figure 3(b)). However, DiI-labeled large-sized neurons (Figure 3(a); cell #1) did not respond to capsaicin without TRPV1 and Na_v_1.8 mRNAs. TRPA1 mRNAs were also detected with TRPV1 and Na_v_1.8 mRNAs in small-sized TG neurons, but maresin 1 (0.35 nM) failed to inhibit TRPA1 currents (Figure 3(a); cell #5).

### 3.4. Maresin 1 Inhibits TRPV1-Evoked Enhancement in Synaptic Transmission and TMJ Inflammation-Induced Synaptic Plasticity in the Trigeminal Nucleus

**Figure 4:**
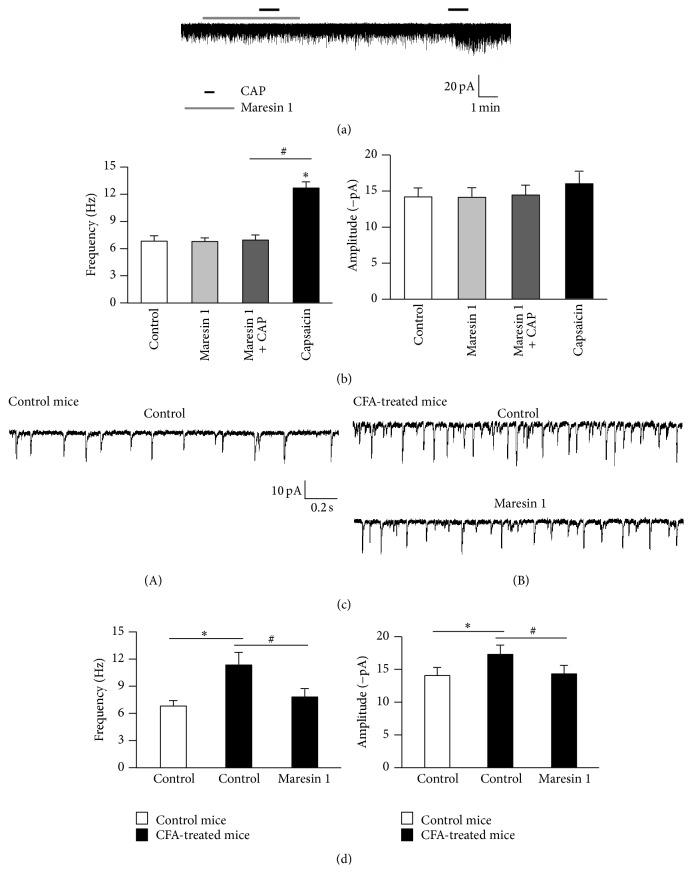
Maresin 1 abolishes capsaicin- (CAP-) induced enhancement in synaptic transmission and TMJ inflammation-induced synaptic plasticity in the trigeminal nucleus. (a) Spontaneous excitatory postsynaptic currents (sEPSCs) traces in Sp5C lamina II neurons of trigeminal nuclei slices before and after capsaicin (500 nM) and maresin 1 perfusion (0.35 nM). (b) Maresin 1 (0.35 nM) blocked capsaicin-induced sEPSC frequency increase (^*∗*^ *p* < 0.05, versus pretreatment baseline, *n* = 8, *t*-test). ((c) (A and B)) Traces of sEPSCs in a trigeminal nuclei slice from control and complete Freund's adjuvant- (CFA-) inflamed (1 day) mice showing sEPSC increases after TMJ inflammation and its inhibition by maresin 1 (0.35 nM). (d) sEPSC frequency and amplitude in the control and CFA-inflamed conditions and the actions of maresin 1 (^*∗*^ *p* < 0.05, compared to noninflamed control; ^#^ *p* < 0.05, compared to CFA-inflamed control, *n* = 5, two-way ANOVA).

To define the functional role of maresin 1 in TMJ pain control, I examined the action of maresin 1 on basal and evoked synaptic transmission in lamina II dorsal horn neurons of the caudal part of the spinal trigeminal nucleus (Sp5C), in which the nociceptive primary afferents form the first intracranial synapses in the trigeminal system. Patch clamp recording in lamina II neurons of Sp5C slices showed that maresin 1 did not alter basal synaptic transmission: both the frequency and the amplitude of spontaneous excitatory postsynaptic currents (sEPSCs) were unaltered after maresin 1 treatment (0.35 nM, Figures 4(a) and 4(b)). Application of capsaicin (500 nM), a selective TRPV1 agonist, to trigeminal nuclei slices evoked a significant increase in the frequency but not amplitude of sEPSCs in Sp5C lamina II neurons [28, 29] (Figures 4(a) and 4(b)). Of interest, maresin 1 (0.35 nM) completely blocked the sEPSC frequency increase by capsaicin (Figures 4(a) and 4(b)), suggesting that maresin 1 can also abolish TRPV1-evoked synaptic plasticity in the Sp5C via possible presynaptic mechanisms. Next, I investigated whether TMJ injury after complete Freund's adjuvant (CFA) injection increased the frequency and amplitude of sEPSCs and further tested whether maresin 1 can reverse these EPSC changes. TMJ inflammation by CFA elicited dramatic increases in both sEPSC frequency (64%) and amplitude (22%) in lamina II neurons of Sp5C slices prepared from inflamed mice (1 day) (Figures 4(c) and 4(d)). Superfusion of the Sp5C slices with maresin 1 at a very low concentration (0.35 nM) reversed these increases in sEPSC frequency and amplitude (Figures 4(c) and 4(d)), indicating that maresin 1 could modulate synaptic plasticity via both presynaptic (sEPSC frequency) and postsynaptic (sEPSC amplitude) mechanisms.

## 4. Discussion

In summary, these results demonstrate that (a) maresin 1, at a very low concentration (0.35 nM), completely blocked capsaicin-induced TRPV1 currents in TMJ-related nociceptive TG neurons, (b) maresin 1 inhibited TRPV1-induced increases in sEPSC frequency in the trigeminal nucleus (Sp5C), and (c) TMJ inflammation by CFA injection induced a greater increase in sEPSCs in the Sp5C, which was abolished by maresin 1. Thus, I propose that maresin 1 can effectively attenuate TMJ inflammation-induced orofacial pain, by modulating TRPV1 function in TMJ-related trigeminal nociceptive neurons and synaptic plasticity in the trigeminal nuclei.

The mechanisms of TMJ pain in the trigeminal system can be different from those of arthritis pain in the somatic system. Although there are anatomic and functional similarities between the spinal and trigeminal somatosensory systems [30], the segmental distribution of the somatic sensory input is less well organized in the trigeminal sensory system. In addition, the distance between the ganglion and its target in the trigeminal system is much shorter than that in the somatosensory system. These differences may enable the trigeminal system to generate greater responses to inflammatory insults [31]. Indeed, my pilot study showed that CFA injection into TMJ induced a greater increase in excitatory synaptic transmission in the trigeminal nucleus (Sp5C) than that induced in the spinal cord by CFA injection into the hind paw of mice (data not shown).

Using an unbiased LC-MS-MS based lipidomics approach, Dr. Serhan's group uncovered two families of endogenous lipid mediators, including resolvins (e.g., resolvin E1, resolvin D1, and resolvin D2) and protectins (e.g., protectin D1 or neuroprotectin D1) in resolving inflammatory exudates [18]. They are biosynthesized from omega-3 fatty acids such as eicosapentaenoic acid (EPA) and DHA and show remarkable potency in treating inflammation-related diseases in animal models [32, 33]. Recent studies have demonstrated that resolvins and protectin potently inhibit somatic inflammatory pain in part by modulating TRPV1 and TRPA1 activity in dorsal root ganglion (DRG) neurons [33–36] in the somatosensory system. Maresin 1 is a newly identified macrophage-derived mediator of inflammation resolution and demonstrates potent anti-inflammatory and potent analgesic actions by inhibiting formalin-induced inflammatory pain and chemotherapy-induced neuropathic pain in the somatic system and suppressing TRPV1 activity in DRG neurons [21]. However, the role of maresin 1 in regulating different TRP channels in the trigeminal system remains unexamined. Therefore, I first labeled the dissociated TG neurons innervating the TMJ by TMJ injection of a retrograde tracer, DiI, as an* in vitro* model to study pain-sensing neurons using patch clamp recordings. Interestingly, the proportion of DiI-labeled neurons is relatively higher in small-sized TG neurons, compared with medium- and large-sized TG neurons (Figure 1(c)). Single-cell RT-PCR showed that most DiI-labeled small-sized TG neurons express both nociceptive markers, TRPV1 and Na_v_1.8 mRNAs (Figure 3(b)) [24]. These data strongly support the notion that the majority, but not all, of the DiI-labeled small-sized TG neurons may be considered to be nociceptors. The present study has identified maresin 1 as a highly potent endogenous inhibitor for TRPV1 in DiI-labeled small-sized TG neurons. It is very remarkable that maresin 1, at very low doses (0.04–0.35 nM), blocked capsaicin-induced TRPV1 currents in DiI-labeled small-sized TG neurons (Figure 2(a)). Of interest, the IC_50_ of maresin 1 (0.11 nM) for TRPV1 inhibition in DiI-labeled small-sized TG neurons is lower than that in small-sized DRG neurons (Figure 2(b)), suggesting that maresin 1 is a more potent inhibitor of TRPV1 in the trigeminal sensory system, as compared to the somatosensory system.

The inhibitory signaling mechanisms of maresin 1 are largely unknown in the trigeminal system. At present, specific receptors for maresin 1 are yet to be identified. A common pattern emerging with maresin 1 signaling is the requirement for specific GPCRs [21]. Remarkably, pretreatment of DiI-labeled TG neurons with pertussis toxin (PTX, 0.5 *μ*g/mL) for 18 h completely blocked maresin 1's inhibitory effects on capsaicin-induced TRPV1 currents. My results demonstrated that the inhibitory signaling effects of maresin 1 on TRPV1 are likely to be mediated by activation of specific PTX-sensitive/G*α*i-coupled GPCRs (Figure 2(d)) in DiI-labeled small-sized TG neurons. These findings suggest a peripheral mechanism of maresin 1 analgesia in the trigeminal system. I also demonstrated that capsaicin (500 nM) substantially increased the frequency of sEPSCs (Figures 4(a) and 4(b)) but not the amplitude of sEPSCs in lamina II neurons of the trigeminal nucleus, indicating that a TRPV1 agonist potentiated glutamate release from presynaptic terminals of primary afferents in the trigeminal system [28, 29]. Notably, the capsaicin-induced increases in sEPSC frequency were blocked by maresin 1 (Figures 4(a) and 4(b)). Thus, maresin 1 may serve as a potent endogenous inhibitor of TRPV1 in the trigeminal system.

Tissue injury-induced spinal cord synaptic plasticity (i.e., central sensitization) contributes greatly to the development and maintenance of chronic pain [37, 38]. Inflammation of the TMJ region triggers the pathogenesis of persistent TMJ pain [4], because of increased synaptic transmission in the trigeminal nuclei including the subnucleus caudalis (Sp5C) [6, 29]. The superficial layers of the Sp5C (laminae I-II) are a region of the brain stem and are highly selective for nociceptive processing [31]. In this study, I showed that TMJ inflammation by complete Freund's adjuvant (CFA) injection elicited dramatic increases in both sEPSC frequency and amplitude in trigeminal nucleus slices prepared from inflamed mice (1 day), as compared to those from control mice (Figures 4(c) and 4(d)). Of note, maresin 1 (0.35 nM) abolished TMJ inflammation-induced increases in sEPSC frequency and amplitude (Figures 4(c) and 4(d)), indicating that maresin 1 could reduce TMJ-inflammatory pain via modulation of synaptic plasticity in the trigeminal system.

These findings demonstrate the potent actions of maresin 1 in regulating TRPV1 function in TMJ-related trigeminal nociceptive neurons and TMJ inflammation-induced synaptic plasticity in the trigeminal nucleus. Therefore, these new findings suggest that maresin 1 may serve as a novel endogenous inhibitor for treating TMJ-inflammatory pain in the orofacial region.

## Figures and Tables

**Table 1:** List of DNA primer sequences designed for single-cell RT-PCR.

Target gene (product length)^a^		Outer primers	Inner primers	Genbank number
TRPV1 (273 bp, 203 bp)	Forward	TGATCATCTTCACCACGGCTG	AAGGCTTGCCCCCCTATAA	NM_001001445.1
	Reverse	CCTTGCGATGGCTGAAGTACA	CACCAGCATGAACAGTGACTGT	

Na_v_1.8 (316 bp, 203 bp)	Forward	CATGACAGAGGAGCAGAAGAAG	CTTTGAATAAGTACCAGGGCTTC	NM_001205321.1
	Reverse	CCAGCCGTTGGTGAAGTAATA	GAACATCTTCATCACACACTCG	

TRPA1 (371 bp, 303 bp)	Forward	GGCTTTTGGCCTCAGCTTTTAT	ATGCCTTCAGCACCCCATT	NM_177781.4
	Reverse	ACACGATGGACCTCTGATC	TGCGTAAGTACCAGAGTGGCAG	

GAPDH (367 bp, 313 bp)	Forward	AGCCTCGTCCCGTAGACAAAA	TGAAGGTCGGTGTGAACGAATT	XM_001473623.1
	Reverse	TTTTGGCTCCACCCCTTCA	GCTTTCTCCATGGTGGTGAAGA	

(*n*, *n*)^a^ indicates product size obtained from outer and inner primers, respectively.
